# Review of juxtaglomerular cell tumor with focus on pathobiological aspect

**DOI:** 10.1186/1746-1596-6-80

**Published:** 2011-08-26

**Authors:** Naoto Kuroda, Hiroko Gotoda, Chisato Ohe, Shuji Mikami, Keiji Inoue, Yoji Nagashima, Fredrik Petersson, Isabel Alvarado-Cabrero, Chin-Chen Pan, Ondrej Hes, Michal Michal, Zoran Gatalica

**Affiliations:** 1 Department of Diagnostic Pathology, Kochi Red Cross Hospital, Shin-honmachi 2-13-51, Kochi City, Kochi 780-8562, Japan; 2 Department of Pathology, Sapporo Kosei General Hospital, 8-5 Kita 3-jo Higashi, Sapporo, Hokkaido 060-0033, Japan; 3 Department of Pathology, Kansai Medical University, Hirakata Hospital, 2-3-1 Shinmachi, Hirakata, Osaka 573-1191, Japan; 4 Division of Diagnostic Pathology, Keio University Hospital, 35 Shinanomachi, Shinjuku-ku, Tokyo 160-8252, Japan; 5 Department of Urology, Kochi Medical School, Kochi University, Kohasu, Oko-cho, Nankoku City, Kochi 783-8505, Japan; 6 Department of Molecular Pathology, Yokohama City University Graduate School of Medicine, B252, 3-9 Fukuura, Kanazawa-ku, Yokohama City, Kanagawa 236-0004, Japan; 7 Department of Pathology, National University Hospital System, 119 074 Singapore, Singapore; 8 Department of Pathology, Mexican Oncology Hospital, Instututo Mexicano del Seguro Social, Mexico City, Mexico; 9 Taipei Veterans General Hospital, No.201, Shi-Pai Rd. Sec2, Taipei 11217, Taiwan; 10 Sikl's Department of Pathology, Charles University Hospital Plzen, Alej Svobody 80, 30460 Plzen, Czech Republic; 11 Depertment of Pathology, Creighton University School, Omaha, NE 68131, USA

## Abstract

Juxtaglomerular cell tumor (JGCT) generally affects adolescents and young adults. The patients experience symptoms related to hypertension and hypokalemia due to renin-secretion by the tumor. Grossly, the tumor is well circumscribed with fibrous capsule and the cut surface shows yellow or gray-tan color with frequent hemorrhage. Histologically, the tumor is composed of monotonous polygonal cells with entrapped normal tubules. Immunohistochemically, tumor cells exhibit a positive reactivity for renin, vimentin and CD34. Ultrastructurally, neoplastic cells contain rhomboid-shaped renin protogranules. Genetically, losses of chromosomes 9 and 11 were frequently observed. Clinically, the majority of tumors showed a benign course, but rare tumors with vascular invasion or metastasis were reported. JGCT is a curable cause of hypertensive disease if it is discovered early and surgically removed, but may cause a fatal outcome usually by a cerebrovascular attack or may cause fetal demise in pregnancy. Additionally, pathologists and urologists need to recognize that this neoplasm in most cases pursues a benign course, but aggressive forms may develop in some cases.

## Introduction

Juxtaglomerular cell tumor (JGCT) is a very rare cause of hypertension that was first described by Robertson et al. in 1967 and the name was coined by Kihara in 1968 [1,2]. To date, approximately 100 cases with JGCT have been reported. Clinically, this tumor is characterized by hypertension, hyperaldosteronism and hypokalemia secondary to excessive renin secretion by tumor cells [3-8]. In this article, we introduce the general overview of JGCT with focus on pathobiological aspects.

### Epidemiology

This tumor affects adolescents and young adults, with peak prevalence in the second and third decades of life with a female predominance [8-10]. Haab et al. (1995) detected eight JGCTs among 30,000 patients at a hypertensive clinic [11].

### Clinical findings

Patients with JGCT present with various symptoms including headaches, retinopathy, double vision, dizziness, nausea, vomiting, polyuria and proteinuria [8,12]. Most of these symptoms may be attributed to hypertension or hypokalemia. Clinically, JGCT is subdivided into three categories [13]. The typical variant, which accounts for the majority of JGCT, has characteristically high renin concentration, hyperaldosteronism, hypokalemia and hypertension [4,5]. Second most common presentation is the atypical variant showing hypertension with normal potassium level [8]. The third, non-functioning variant is very rare and is characterized by a normal blood pressure and normal potassium level [14-16]. Clinicians should strongly suspect JGCT if they encounter adolescent or young adult patients with severe or even moderate hypertension associated with an unexplained secondary hyperaldosteronism [6]. JGCT may cause malignant hypertension [17]. A case of JGCT associated with membranous glomerulonephritis was also reported [18].

### Radiological findings

Ultrasonography of the kidneys usually shows a hypoechoic mass [19,20]. Computed tomography (CT) scan is very useful for the detection of renal tumor, even when other imaging analyses is negative [8,21]. CT scan shows finding of low, low-to iso- or low- to high-density without enhancement [20,22-24]. In dynamic CT scan, JGCT is not stained during early phase, but stains moderately during late phase after contrast enhancement [24-26]. Magnetic resonance imaging is also a powerful diagnostic procedure [12,23,25]. MRI shows an iso-signal intensity area on T1-weighted images and a high-signal intensity area on T2-weighted images, but MRI findings seem to variable [23,26,27].

### Pathological findings

#### Macroscopic findings

Grossly, the tumor is well circumscribed and complete or partial fibrous capsule is observed in most cases [9,28]. The cut surface of the tumor imparts yellow to gray-tan in color with frequent hemorrhage [9]. The tumor is usually small solitary and unilateral, but the tumors exceeding 3 cm are occasionally observed [8,29,30]. A case of extrarenal JGCT arising in bone has been reported [31].

#### Microscopic findings

**Figure 1:**
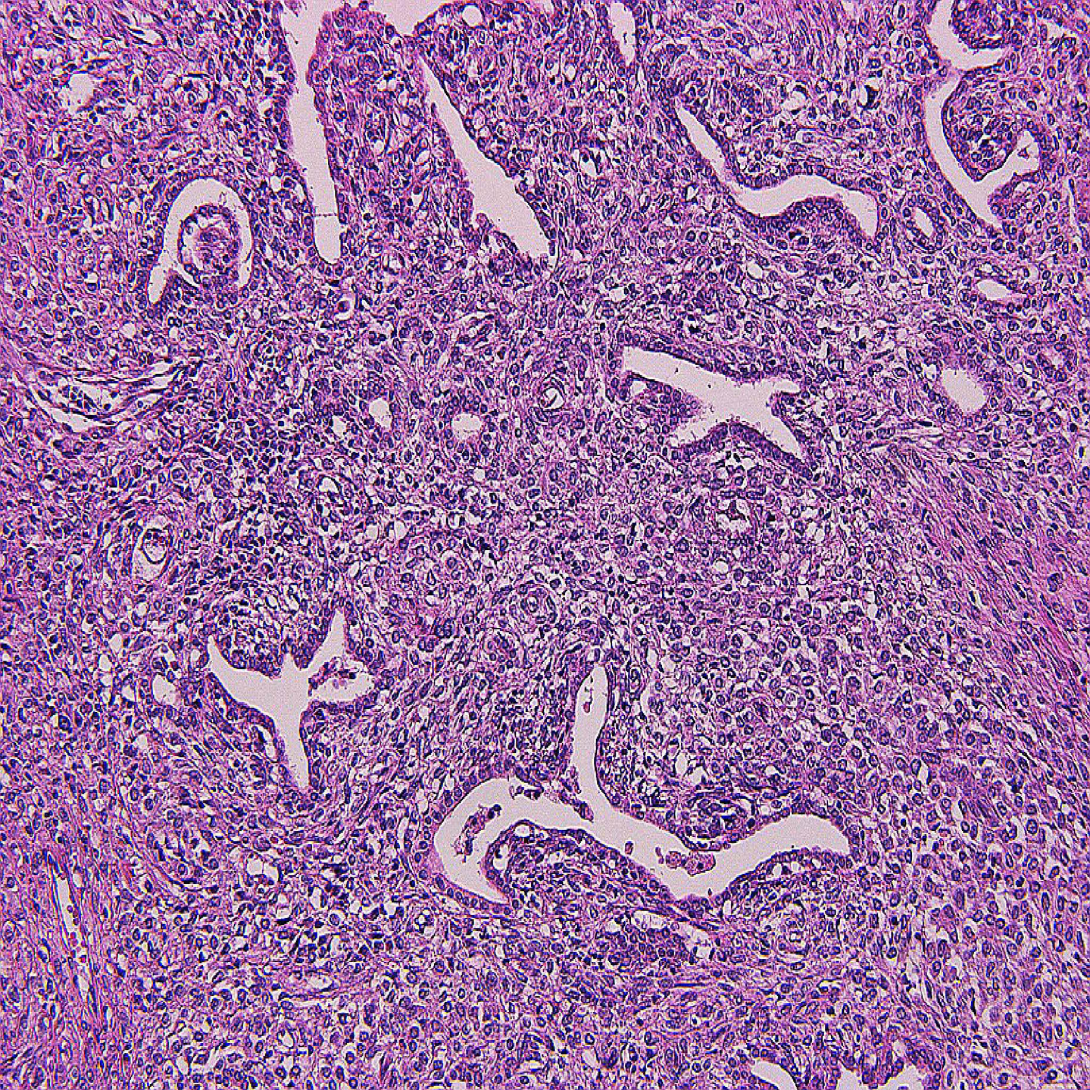
**Microscopic findings**. Low power view. Neoplastic cells with ovoid to polygonal in shape proliferate with growth pattern of solid sheets. Intervening tubular component is also seen.

**Figure 2:**
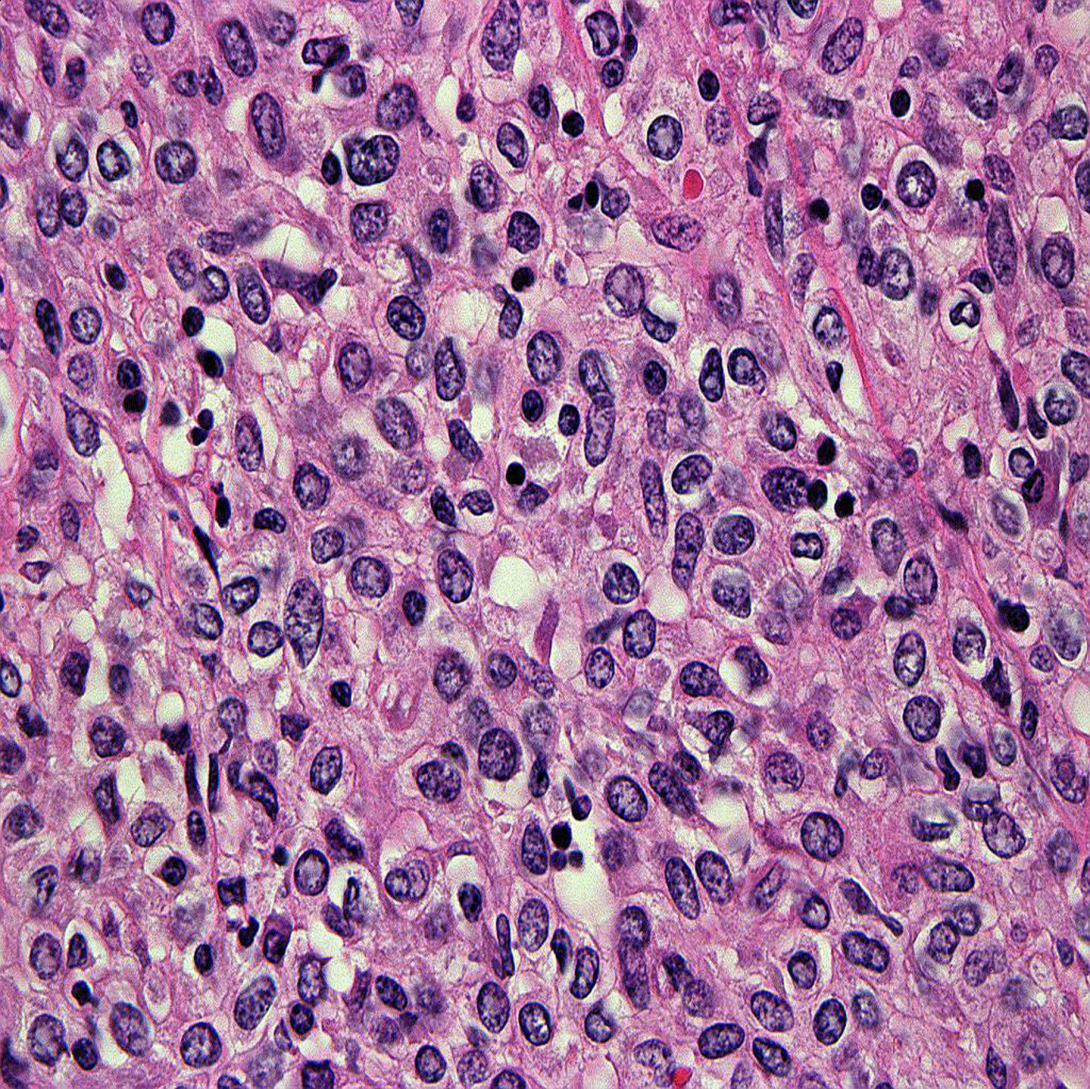
**Microscopic findings**. High power view. Cell border is generally indistinct and mitotic activity is absent. There is no or little pleomorphism of nuclei, but multinuclear cells are focally seen.

**Figure 3:**
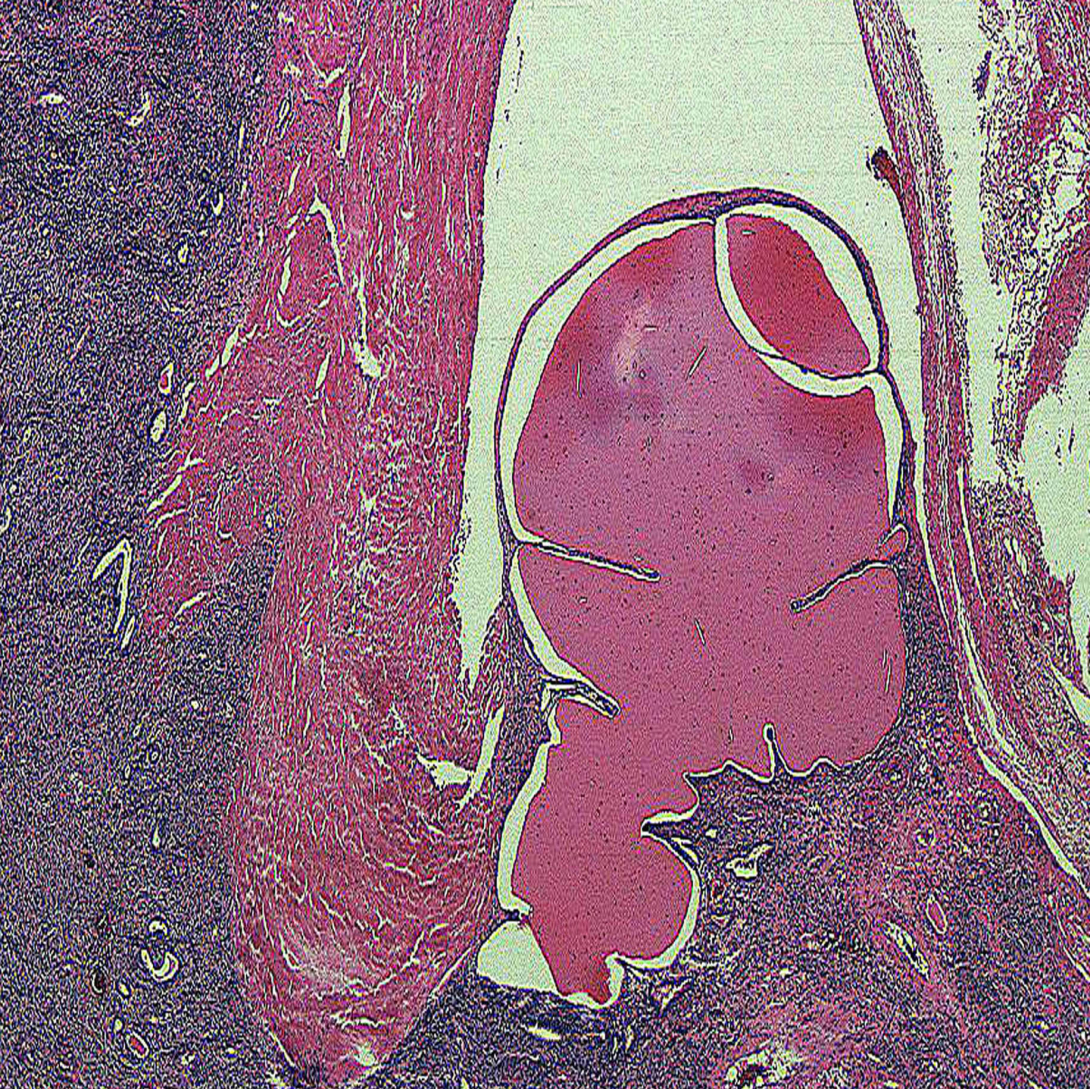
**Microscopic findings**. Vascular invasion is seen.

Histologically, the tumor consists of solid sheets of closedly packed uniform round to polygonal cells with oval to round nuclei, pale to eosinophilic cytoplasm, inconspicuous nucleoli and indistinct cell border (Figure 1, 2)[10]. Microcystic pattern is also seen [9,32,33]. Focal moderate nuclear atypia may be seen, but mitotic figures are generally absent [9]. Entrapped tubules are identified; predominantly at the peripheral area of the tumor but tubular cells suggestive of neoplastic nature has been reported [28,29,34,35]. Thin-wall and thick-wall blood vessels with hyalinization are intervened within the tumor and antler vascular pattern simulating hemangiopericytoma may be noted [3,9,28,32,35]. Mast cells often infiltrate in the stroma [4,36]. Penetration of fibrous capsule or vascular invasion by tumor cells may be observed in some cases [37-39](Figure 3).

#### Histochemical findings

Intracytoplasmic granules suggestive of renin storage may be detected by Bowie, periodic acid-Aschiff, and phostotungstic acid-hematoxylin-azan stains [2-4,28,29,40-42]. Toluidine blue stain is useful in the detection of mast cells in the stroma [43].

#### Immunohistochemical findings

**Figure 4:**
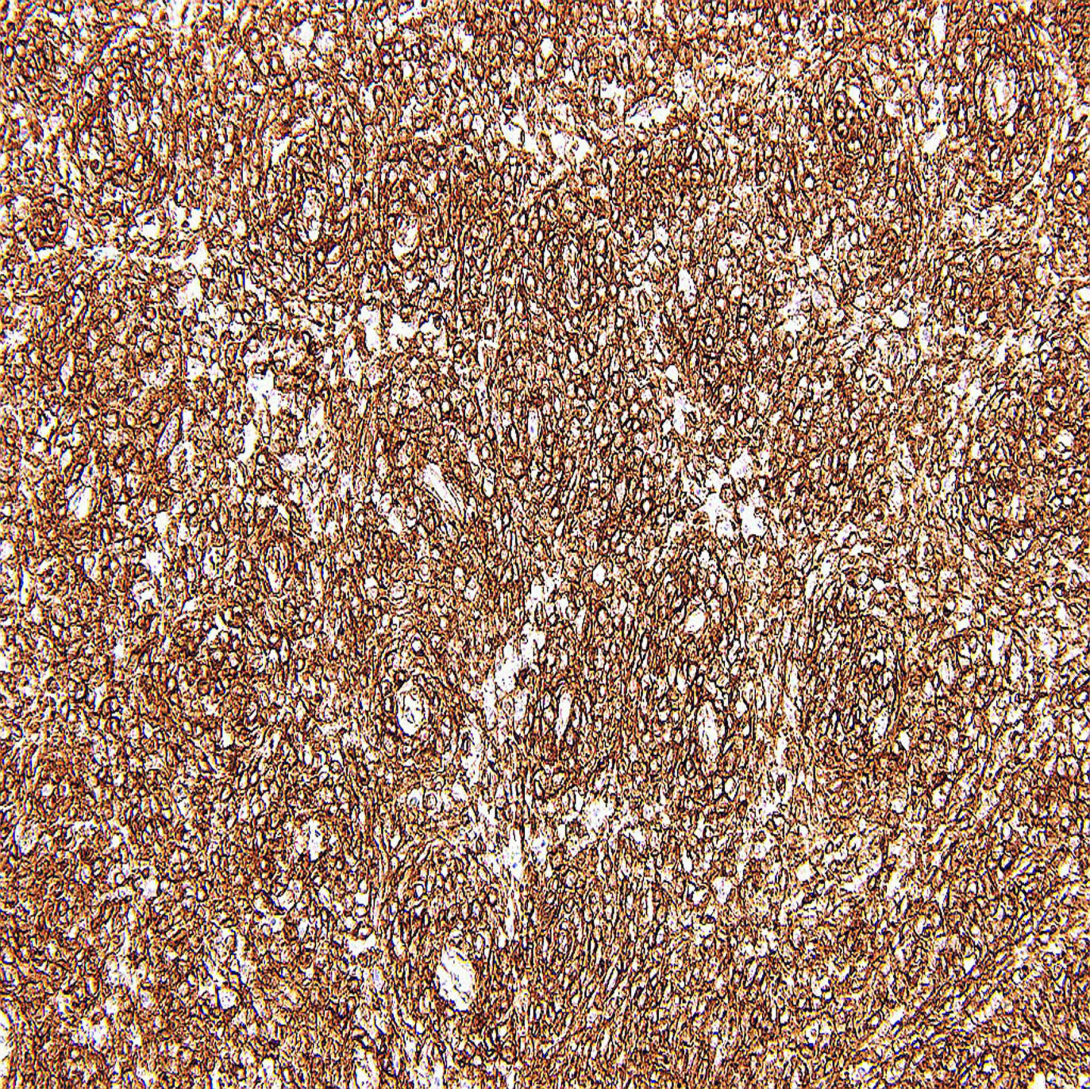
**Immunohistochemical findings**. Tumor cells show the labeling for CD34.

The diagnosis is confirmed by positivity for renin in the cytoplasm [12,21,22,34,35,42]. However, renin positivity may be observed in some cases of Wilms tumor, RCC or renal oncocytoma [7]. Most neoplastic cells are diffusely immunoreactive for vimentin and CD34 [44](Figure 4). The immunoreactivity to smooth muscle actin and CD117 varies from case to case [9,10]. Tumor cells demonstrate no reactivity to von Willebrand factor, CD31, desmin, S-100 protein, melanosome-related antigen, chromogranin A, synaptophysin, and neuron specific enolase [9,35]. Epithelial components are positive for cytokeratin CAM5.2 and cytokeratin 7 [35].

#### In situ hybridization findings

Messenger RNA of renin is found in the cytoplasm of tumor cells of JGCT. Some investigators suggest that renin synthesis and storage may be concordant [45], whereas others consider that the positivity of messenger RNA in the tumor cytoplasm may be due to uptake of renin rather than renin production [37].

#### Ultrastructural findings

The cytoplasm of tumor cells contains characteristic rhomboid-shaped renin protogranules, abundant rough endoplasmic reticulum and prominent Golgi apparatus [4,9,10,22,29,34,40-42,44,46]. Secretory granules of various size and shapes are also observed [36]. Micropinocytotic vesicles and submembranous plaques suggestive of smooth muscle differentiation may be also contained in the cytoplasm [32,47]. The presence of nerve fibers have been reported [48]. Ultrastructural immunocytochemistry showed that immunoreactive renin is observed not only in crystalline protogranules or round membrane bound granules but also in intermediate form [49].

### Differential Diagnosis

The distinction from glomus tumor, hemangiopericytoma, metanephric adenoma, papillary RCC, collecting duct carcinoma (CDC), urothelial carcinoma (UC), renal epithelioid angiomyolipoma (AML) and Wilms tumor should be considered. JGCT may possess the growth pattern reminiscent of glomus tumor. However, glomus tumor contains no epithelial component and is immunohistochemically negative for renin. Hemangiopericytoma lacks polygonal cells and thick-walled vessels. Solitary fibrous tumor often shows the hemangiopericytomatous pattern, but this tumor is frequently reactive for CD99 and bcl-2. JGCT with prominent papillary growth pattern may resemble papillary RCC [35]. However, papillary RCC lacks biphasic pattern of round to polygonal cells and epithelial cells. Metanephric cells typically show acinar growth pattern and psammoma bodies are often seen in the stroma. Solid sheets growth pattern of JGCT may evoke the diagnosis of CDC or poorly differentiated UC. However, CDC or urothelial carcinoma is macroscopically located in the renal medulla or hilar region, respectively, but the location of many JGCTs is the renal cortex. Renal angiomyolipoma may contain minor adipose tissue component and show the immunoreactivity for melanosome-related antigen. Wilms' tumor typically contains blastemal cells showing nuclear overlapping.

### Molecular genetic findings

There are only a few limited reports on genetic abnormalities of JGCT. Using karyotype, comparative genomic hybridization (CGH) and interphase fluorescence *in situ *hybridization (FISH), the gain of chromosome 10 as well as losses of chromosomes 9 and X and most of chromosome 11q may be important pathogenetic events in JGCT [50]. One case demonstrated monosomy of chromosomes X, 6, 9, 11, 15 and 21 using FISH analysis [51]. Two tumors revealed losses of chromosomes 9 and 11 by CGH [52]. Aneuploid karyotype and complex genomic imbalance observed in two cases may reflect a possible development for local recurrence or distant metastasis, namely uncertain malignant potential [50,52].

### Therapy

The complete tumor resection by radical or partial nephrectomy is the best modality for JGCT [35,53]. Antihypertensive agents should be treated for hypertension until accurate diagnosis is made, but blood pressure, plasma renin level usually normalize after the nephrectomy in most cases with JGCT [2,23,25,54-56]. However, hypertension may continue because of hypertensive angiopathy even after the complete tumor removal in approximately 10% of all cases [10,25,47].

### Prognosis

The majority of cases with JGCT have behaved in a benign manner and neither local recurrence nor metastasis has occurred with either radical or partial nephrectomy. However, one metastatic case with JGCT has been reported to date [38]. Additionally, a case of JGCT that caused death due to massive brain hemorrhage secondary to severe hypertension has been reported [57]. A case of JGCT causing fetal demise has been noted [58].

## Conclusion

Since the discovery of this tumor approximately 40 years ago, many common histological features including histochemistry, immunohistochemistry and ultrastructure have been elucidated and, as a result, JGCT gained the status as the distinct mesenchymal tumor entity from other renal tumor. However, there are only a few genetic studies of JGCT because of the rarity of this disease. As we encountered a case of JGCT with vascular invasion, the true biological behavior of JGCT will be needed to be elucidated. Accordingly, genetic features of JGCT need to be clarified by the future investigations.

## Authors' contributions

NK, HG, SM and YN describe pathological findings, KI write epidemiology and clinical findings, FP, IAC and CCP describe molecular genetic findings, and CO, OH, MM and ZG revise the manuscript. All authors read and approved the final manuscript.

## Competing interests

The authors declare that they have no competing interests.
